# Student perspectives on the diversity climate at a U.S. medical school: the need for a broader definition of diversity

**DOI:** 10.1186/1756-0500-6-154

**Published:** 2013-04-17

**Authors:** Jasmeet S Dhaliwal, Lori A Crane, Morgan A Valley, Steven R Lowenstein

**Affiliations:** 1 University of Colorado School of Medicine, Aurora, Colorado, USA; 2 Department of Community and Behavioral Health, Colorado School of Public Health, Aurora, Colorado, USA; 3 Department of Emergency Medicine, University of Colorado School of Medicine, Aurora, Colorado, USA

**Keywords:** Diversity climate, Diversity, Medical student perspectives, Medical education

## Abstract

**Background:**

Medical schools frequently experience challenges related to diversity and inclusiveness. The authors conducted this study to assess, from a student body’s perspective, the climate at one medical school with respect to diversity, inclusiveness and cross-cultural understanding.

**Methods:**

In 2008 students in the doctor of medicine (MD), physical therapy (PT) and physician assistant programs at a public medical school were asked to complete a diversity climate survey consisting of 24 Likert-scale, short-answer and open-ended questions. Questions were designed to measure student experiences and attitudes in three domains: the general diversity environment and culture; witnessed negative speech or behaviors; and diversity and the learning environment. Students were also asked to comment on the effectiveness of strategies aimed at promoting diversity, including diversity and sensitivity training, pipeline programs, student scholarships and other interventions. Survey responses were summarized using proportions and 95 percent confidence intervals (95% CI), as well as inductive content analysis.

**Results:**

Of 852 eligible students, 261 (31%) participated in the survey. Most participants agreed that the school of medicine (SOM) campus is friendly (90%, 95% CI 86 to 93) and welcoming to minority groups (82%, 95% CI 77 to 86). Ninety percent (95% CI 86 to 93) found educational value in a diverse faculty and student body. However, only 37 percent (95% CI 30 to 42) believed the medical school is diverse. Many survey participants reported they have witnessed other students or residents make disparaging remarks or exhibit offensive behaviors toward minority groups, most often targeting persons with strong religious beliefs (43%, 95% CI 37 to 49), low socioeconomic status (35%, 95% CI 28 to 40), non-English speakers (34%, 95% CI 28 to 40), women (30%, 95% CI 25 to 36), racial or ethnic minorities (28%, 95% CI 23 to 34), or gay, lesbian, bisexual or transgendered (GLBT) individuals (25%, 95% CI 20 to30). Students witnessed similar disparaging or offensive behavior by faculty members toward persons with strong religious beliefs (18%, 95% CI 14 to 24), persons of low socioeconomic status (12%, 95% CI 9 to 17), non-English speakers (10%, 95% CI 6 to 14), women (18%, 95% CI 14 to 24), racial or ethnic minorities (12%, 95% CI 8 to 16) and GLBT individuals (7%, 95% CI 4 to 11). Students’ open-ended comments reinforced the finding that persons holding strong religious beliefs or conservative values were the most common targets of disparaging or offensive behavior.

**Conclusions:**

These data suggest that medical students believe that diversity and a climate of inclusiveness and respect are important to a medical school’s educational and clinical care missions. However, according to these students, the institution must embrace a broader definition of diversity, such that all minority groups are valued, including individuals with conservative viewpoints or strong religious beliefs, the poor and uninsured, GLBT individuals, women and non-English speakers.

## Background

There is widespread agreement in medical education that a diverse student body and faculty enhance the educational experience for medical students. Numerous expert opinions and empiric studies suggest that diversity strengthens the learning environment, improves learning outcomes and helps prepare students to care for an increasingly diverse population [1-9]. There is also evidence that improving diversity in medical schools may help to reduce health disparities [10] by improving communication and patient care outcomes [3,11,12], increasing the number of physicians willing to practice in underserved areas [1,13-15], and inspiring more innovative problem solving and a broader research agenda [16-18]. This strong body of evidence provides support for recent Supreme Court decisions related to race-conscious admissions [19] and for ongoing efforts by medical schools, the Institute of Medicine [20] and the Association of American Medical Colleges (AAMC) [21] to improve diversity in the health professions.

While there are clear benefits to medical school diversity, the task of improving diversity in medical education is complicated by a variety of financial, legal, educational and recruitment-related challenges. In addition, there is no consensus regarding the best definition of “diversity”. The AAMC and most medical schools agree that race, ethnicity, socioeconomic status, sexual orientation and geography are essential elements of diversity [22]. However, each medical school is expected to develop its own operational definition of diversity, after considering the school’s history and traditions, geographic locale, community responsibilities and educational, clinical, research and service missions. It is not known how often students’ perspectives are considered when school-specific definitions of diversity are developed.

In 2007 the faculty and administration of the University of Colorado School of Medicine (SOM) adopted a broad definition of diversity embracing “race, ethnicity, gender, religion, socioeconomic status, sexual orientation and disability”. The definition of diversity also includes “life experiences, record of service and employment and other talents and personal attributes that can enhance the scholarly and learning environment” [23]. The purpose of this study was to assess the school’s diversity climate, and thereby indirectly assess the appropriateness of the institution’s established definition of diversity, from the students’ point of view.

We asked every student enrolled in the doctor of medicine (MD), physical therapy (PT) and child-health associate/physician assistant (CHA/PA) programs at the University of Colorado SOM to complete an online survey about diversity. The SOM is a public medical school within the University of Colorado system.

## Methods

### Survey design

The survey was composed of 24 Likert-scale, short-answer and free-text questions. The majority of the questions were derived from published survey instruments [24-28]. All questions were pilot-tested among student and faculty colleagues to improve the clarity of the questions and response categories. This process served as the validation instrument for the survey. The survey included questions to assess student experiences and attitudes in three principal domains:

(1) General environment and culture: How do students perceive the climate and culture within and outside the classroom? For example, is the SOM welcoming to people from minority groups? Is it sexist, racist or homophobic?

(2) Witnessed negative speech or behaviors: Have students witnessed other students, residents or faculty members make disparaging remarks or engage in offensive or intimidating behaviors toward members of minority groups? If so, which minority groups are most frequently targeted?

(3) Diversity and the learning environment: Do students feel that learning is enhanced by having a student body and faculty who represent diverse backgrounds?

In addition, the survey contained several demographic questions that asked students to identify their gender, academic program (MD, PT or CHA/PA programs), religious affiliation and racial and ethnic minority status. We also asked students to comment on the perceived effectiveness of a variety of strategies aimed at promoting diversity and inclusiveness, including recruitment of a more diverse faculty, student scholarships, mentoring for minority students, diversity and sensitivity training, workshops and other interventions.

For the majority of questions, we asked students whether they agreed or disagreed with a statement (for example, “The School of Medicine is welcoming to people from minority groups”). Each statement was followed by four Likert-scale response options, ranging from 1 (“strongly disagree”) to 4 (“strongly agree”). Open-ended prompts asking for examples or more detailed information followed many of the Likert-scale questions.

With respect to “witnessed negative speech or behaviors”, we asked students if they had “ever witnessed residents or fellow students make disparaging remarks or engage in offensive, hostile or intimidating behaviors toward members of minority groups”. Seven groups were listed: racial or ethnic minorities; persons with disabilities; women; people with strong religious beliefs; people who speak English as a second language; people of low socioeconomic status; and people who are gay, lesbian, bisexual or transgendered. For each minority group, students responded by choosing one of four options: “no, never”; “yes, on one occasion”; “yes, a few times”; or “yes, frequently”. A similar question addressed disparaging remarks or offensive behaviors made by faculty members. Participants were then asked an open-ended question regarding the setting and nature of the witnessed speech or behaviors.

### Recruitment of participants

We distributed the online survey between May 21st and June 11th, 2008 to all students enrolled in the School of Medicine’s MD, PT and CHA/PA programs. All students enrolled at the School of Medicine have their email address incorporated into a master email listserv maintained by their respective academic program, and all students on these email lists were contacted by email to participate in the study. A single reminder email was sent one week later. The survey was voluntary and anonymous, and students could leave any question unanswered. The study was approved as exempt by the Colorado Multiple Institutional Review Board prior to data collection (Protocol # 07–0976).

### Data analysis

We calculated proportions and 95 percent confidence limits (95% CI) to summarize survey responses. We also performed bivariate analyses to test for associations between the experiences, attitudes and observations reported by students and two salient demographic attributes (self-identified gender and membership in a minority group). We measured statistical significance using the chi-square test (or Fisher’s Exact Test, where appropriate). To measure the strength of the associations, we calculated odds ratios and 95 percent confidence limits. To facilitate ease of interpretation, we collapsed the ordinal Likert-scale responses into dichotomous categories: “Agree” (which included “strongly agree” and “agree”) and “disagree” (which included “disagree” or “strongly disagree”). For the open-ended questions requiring typed responses, we utilized inductive content analysis, with themes and categories that emerged from the data through careful examination and comparison [29]. We summed the number of comments within each of the derived thematic categories and also included examples from each theme in this report.

## Results

### Description of survey participants

Of the 852 students eligible for the survey, 261 (31%) participated. Question-specific response rates ranged from 73 to 100 percent. The response rate for students in the MD program was 34 percent, compared with only 12 percent for PT and CHA/PA students.

Over half of the participants (58%) were female, and most (87%) were enrolled in the MD program. One hundred ninety-four students (83%) said they were white and non-Hispanic; 20 students (9%) identified themselves as African-American, Hispanic, Native American, Native Alaskan or Hawaiian Native; and 21 students (9%) reported they were members of “another minority group”. Students were asked about their religious affiliation: almost half (48%) reported their religious affiliation as “Christian”; 43 percent reported “no religious affiliation”; and 9 percent said they had a religious affiliation “other than Christian”. Ten students (4%) said they considered themselves members of a minority group due to their sexual orientation.

Based on available demographic data from the School of Medicine, there were no significant differences between the survey participants and the student body-at-large with respect to race, ethnicity, or gender. Seventeen percent of survey participants identified themselves as racial or ethnic minorities, compared with 16 percent among all students in the SOM. Women composed 58 percent of participants, compared with 58 percent among all students in the SOM. Hence, survey participants and non-participants do not appear to differ with respect to race, ethnicity or gender.

### General environment and culture

The majority of students “strongly agreed” or “agreed” that the SOM is friendly (90%; 95% CI 86 to 93) and is welcoming to people from minority groups (82%; 95% CI 77 to 86). Smaller percentages of participants “strongly agreed” or “agreed” that the SOM campus is homophobic (9%; 95% CI 6 to 13), racist (6%; 95% CI 4 to 10) or sexist (7%; 95% CI 5 to 12). When asked whether the SOM itself is “diverse”, only 36 percent (95% CI 30 to 42) of participants agreed. There were no significant differences between the responses of minority and non-minority students with one notable exception. Students who identified themselves as a minority due to their sexual orientation were much more likely to agree that the SOM was “homophobic”, when compared with students who did not identify with this minority group (50%; 95% CI 24 to 76 versus 6%; 95% CI 3 to 10).

### Witnessed negative speech or behaviors

With respect to offensive, hostile or intimidating remarks or behaviors made by fellow students or residents, participants reported witnessing incidents targeting each of the seven minority groups listed (Table 1). “People with strong religious beliefs” were targeted most often, followed by “people of low socioeconomic status” and “people who speak English as a second language”. “Women”, “people from racial or ethnic minority groups”, and “people who are gay, lesbian, bisexual or transgendered” were targeted less often. People with disabilities were least often targeted by fellow students.

**Table 1:** Frequency of witnessed disparaging remarks and offensive, hostile or intimidating behaviors made toward minorities

	**Remarks made by residents or other students**					**Remarks made by faculty**				
	**On one occasion**	**A few times**	**Frequently**	**One or more times**		**On one occasion**	**A few times**	**Frequently**	**One or more times**	
	**% (n)**	**% (n)**	**% (n)**	**% (n)**	**95% CI**	**% (n)**	**% (n)**	**% (n)**	**% (n)**	**95% CI**
People with Strong Religious Beliefs	13.0 (32)	22.7 (56)	6.9 (17)	42.5 (105)	36.5, 48.7	9.5 (23)	6.2 (15)	% (n)2.9 (7)	18.5 (45)	14.1, 23.9
People of Low Socioeconomic Status	14.6 (36)	15.8 (39)	3.6 (9)	34.0 (84)	28.4, 40.1	4.6 (11)	7.1 (17)	0.4 (1)	12.0 (29)	8.5, 16.8
People who Speak English as a Second Language	13.4 (33)	15.8 (39)	4.9 (12)	34.0 (84)	28.4, 40.1	2.9 (7)	5.8 (14)	0.8 (2)	9.5 (23)	6.4, 13.9
Women	11.7 (29)	16.9 (42)	1.2 (3)	29.8 (74)	24.5, 35.8	8.7 (21)	8.3 (20)	1.2 (3)	18.2 (44)	13.8, 23.5
People from Racial or Ethnic Minority Groups	10.5 (26)	16.9 (42)	0.8 (2)	28.2 (70)	23.0, 34.1	5.4 (13)	6.2 (15)	0.0 (0)	11.6 (28)	8.2, 16.3
People who are Gay, Lesbian, Bisexual or Transgendered	11.7 (29)	10.9 (27)	2.0 (5)	24.6 (61)	19.6, 30.3	4.5 (11)	2.1 (5)	0.4 (1)	7.0 (17)	4.4, 10.9
People with Disabilities	8.5 (21)	6.5 (16)	0.4 (1)	15.4 (38)	11.4, 20.4	1.2 (3)	2.9 (7)	0.0 (0)	4.1 (10)	2.3, 7.4

The table lists the percentage and absolute number of affirmative student responses to the question, “Have you ever witnessed fellow students or residents (left columns) or faculty members (right columns) make disparaging remarks or engage in offensive or intimidating behaviors toward members of the following minority groups?” Responses were recorded for each of the 7 minority groups listed and are divided into two sections based on whether perpetrators were identified as students/residents or faculty members.

When students were asked to state whether they had witnessed incidents perpetrated by faculty members, positive responses were far less common (Table 1). But as before, “people with strong religious beliefs” were the most common targets of negative remarks and behaviors; almost one in five students (18%) had witnessed offensive or hostile remarks or behaviors directed toward this group. Similarly, “women”, “people of low socioeconomic status”, “people who speak English as a second language” and “racial or ethnic minorities” were relatively common targets.

**Figure 1:**
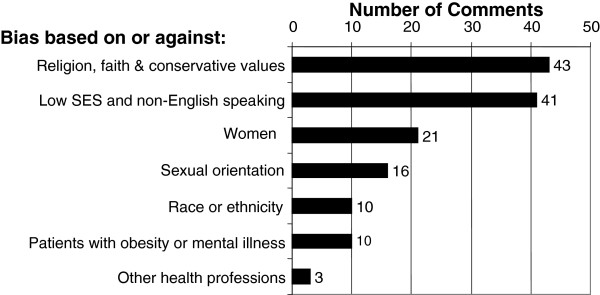
**Categorization and distribution of witnessed remarks and behaviors.** This graph represents the thematic categorization and distribution of student responses about witnessed disparaging remarks and offensive or intimidating behavior directed toward minority groups. Specifically, the graph depicts the number of free-text comments falling into 7 thematic areas derived de novo after content analysis of open-ended responses to the question, “Please comment on the nature of [any witnessed negative] remarks or [offensive or intimidating] behaviors and the context in which they took place. For example, were the remarks made in the classroom or clinical setting? Were the remarks or behaviors directed towards patients, students, staff, faculty or someone else? Who (student or resident) was responsible for the remarks or behaviors?”.

The open-ended responses revealed a similar theme. According to the free-text descriptions and examples provided by the students, individuals demonstrating “strong religious faith or conservative values” were the most common targets of disparaging remarks (Figure 1). Forty-three (25%) of the 174 comments pertained to this thematic category. As with the Likert-scaled questions, bias against low socioeconomic status individuals, non-English speakers and women were also mentioned frequently. Of note, students mentioned that these incidents had occurred in all campus settings, including classrooms, small group seminars, clinics and the wards. Comments targeting people of low SES and non-English speakers generally pertained to hospitalized patients. Representative comments are included in the following section, List of Representative Comments on Witnessed Remarks and Behaviors.

#### List of representative comments regarding witnessed remarks and behaviors

The following is a list of representative comments from an open-ended prompt asking students to clarify the nature and setting of witnessed negative remarks and behaviors toward minorities. The selected comments are from the four thematic areas most often discussed by students.

##### Religion, faith and conservative values

It is disrespectful to imply that belief in a higher being or creator is ignorant, especially when there are many prominent scientists who share such beliefs. I would like to think that by the time we are in medical school, lecturers would respect the fact that there are people who disagree with them and not feel the need to use their platform as a soapbox for disparaging those whose religious or political views may differ from their own.

One professor in first year drew a blastocyst on the board and erased it. Then he suddenly covered his head as if to protect himself and said, “Uh-oh, the anti-abortionists are going to hurt me for erasing that”.

The remarks that have been the most personally disturbing to me have been the complete lack of respect towards people who have religious beliefs of any kind…

##### Low socioeconomic status and non-english speakers

VA and [community hospital] patients have become synonymous with “low-income”, “substance-abusing”, “non-compliant”, “resource-depleting”, “unappreciative” and, to a lesser extent, “undeserving”. This sentiment is pervasive.

There are general comments about a patient with a certain pathology, and residents and attendings will comment something like "I'll bet they don't have insurance" or "This one's on the hospital" or something like that - without knowing anything about the patient.

Sometimes it seems that people with low incomes do not have the right to complain, because they don’t pay for their care.

##### Bias against women

Comments regarding women, such as “how much easier is for them to get into medical school” and “how they won’t work full-time anyway”.

I was once on a service that had a daily rating scale going of which female on the [floor] had the best [physical appearance].

One comment made by an elderly male attending was directed to the two female students on the team ---- “this field is not ideal for women because we take time off to have babies”.

##### Bias based on sexual orientation

Many of the professors get the terms "sex" and "gender" confused. During one lecture…a professor made some comments that were very, very insensitive to transgendered individuals.

Our school’s [diversity] efforts are so focused on racial and ethnic minorities that other aspects of diversity are being excluded. This is especially true for GLBTI issues.

### Diversity and the learning environment

Students were asked whether they believed their learning was “enhanced by having students and faculty who represent diverse backgrounds”. Almost all (90%) “strongly agreed” or “agreed”. There was no significant difference between the responses of self-identified minority students as compared with non-minorities (91% vs. 89%; p = 0.68).

### Student recommendations to improve the diversity climate

The final series of questions asked students to comment on the school’s diversity climate and to offer recommendations for programmatic improvement. The majority of participants spoke positively about ongoing diversity efforts and the current diversity climate. At the same time, some students offered more critical comments, many asserting that the school’s diversity definition was too narrow. These students argued that demographic characteristics, including race, ethnicity and gender, should be replaced or augmented by characteristics such as life experiences, political and social ideologies, values, family structure and socioeconomic status. For example, one student wrote “the SOM should define diversity based on socio-economic status and history, rather than…race and ethnicity. Those [attributes] are not a true representation of students’ pasts, presents and futures”. Another wrote, “rather than singling individuals out because of race, religion, sexual preference or gender, the SOM ought to promote a climate of acceptance towards all beliefs --- this means creating a culture that does not promote one political or religious view over another”. Some students were also unhappy with the School’s continued focus on what they perceived to be targeted recruitment of racial and ethnic minorities; a few students referred to such programs as “quotas” or “affirmative action”. Students highlighted the importance of pipeline programs in high schools and colleges as the most effective way to recruit underrepresented minorities into the health professions. Students also reported that GLBT issues were not addressed adequately in the curriculum or during public forums about diversity.

## Discussion

In this survey we sought to assess the diversity climate at one medical school, from the student body’s point of view. The results suggest that the majority of MD, PT and PA students perceive the medical school campus to be friendly and welcoming to minority groups. Furthermore, the vast majority of students believe that learning is enhanced by a diverse student body and faculty. At the same time, less than 40 percent of students view their own campus as diverse.

Students also reported that they frequently hear disparaging remarks or witness offensive behaviors targeting minority groups. These incidents occur in classroom, small group and clinical settings. Survey participants were 2–3 times more likely to witness offensive behaviors perpetrated by fellow students or residents than by faculty members. We were surprised to learn that individuals holding or expressing strong, even fundamentalist, religious beliefs and conservative political values are most often targeted during these incidents. This finding highlighted a potential minority group and source of diversity not previously considered in the working definition of diversity at the School of Medicine. Other groups frequently targeted include people of low socioeconomic status [30], non-English speakers, racial and ethnic minorities [31], members of the GLBT community [32] and women [24,33]. While bias against people of low socioeconomic status, non-English speakers, racial and ethnic minorities, members of the GLBT community, and women have been documented at medical schools, to our knowledge this is the first time that bias against individuals with conservative religious and political views has been reported. The students’ comments clearly indicate that these incidents negatively affect the diversity climate and the learning environment at the school. Ultimately, the survey results highlight problems arising within the school’s diversity climate and also suggest that the definition of diversity at the School of Medicine be further broadened. Our study also highlights the utility of querying student perspectives when developing institutional diversity definitions, policies and programming.

### Limitations

While the results of this survey offer important insights into student perceptions of diversity and inclusiveness, there are several important limitations. First, the survey was conducted at a single public medical school (in a Western state), which may limit the generalizability of the results. Second, the survey was voluntary, and the response rate was 31 percent. While survey participants and non-participants did not differ with respect to race, ethnicity or gender, participants may differ from non-participants with respect to their views on diversity and the campus climate. We cannot assess the magnitude or direction of any non-participation bias. Nonetheless, when the survey results were presented to the student body in large group settings, there was a general consensus, by a show of hands, that the survey results were representative of student experiences at the medical school. Moreover, in the context of the diversity climate at medical schools, our novel finding that individuals with strong religious beliefs and conservative political values are often targets of biased behavior is significant irrespective of the response rate, as it is the first time this has ever been reported in any quantitative or qualitative fashion. A third limitation is the relatively small sample. Although the overall sample size (n = 261) allowed us to summarize responses with adequate precision, some subgroup comparisons (for example, responses according to minority status or academic program within the medical school) were not possible due to small sample sizes. Fourth, the survey data do not permit calculation of true rates. For example, our data suggest that that 12 percent of participants report witnessing faculty make disparaging remarks or engage in offensive behaviors toward people who speak English as a second language at least once in the past year. However, we have no information about true “denominators” --- that is the number of encounters between the survey participant and the faculty member, or the number of witnessed encounters between the faculty member and people who speak English as a second language, whether positive or negative.

### The medical school’s responses to the climate survey

Three recommendations emerge from these data. First, medical schools should consider adopting a broader definition of diversity, one that includes religious and spiritual values and political beliefs. Medical schools should be vigilant in ensuring that students with strong religious beliefs and conservative political views are respected. According to the data from this study, the diversity definition must also include women, members of the GLBT community, non-English speakers, the uninsured and the poor. Second, medical school diversity climate surveys should include questions about tolerance and respect for individuals holding strong religious beliefs and conservative social and political values. Third, our study found that students, residents and faculty were all involved; therefore, diversity awareness and training programs must include all members of a medical school’s learning community.

At our institution, the results of this climate survey have been shared with the MD student body, department chairs and faculty senate, and also with the leaders of the undergraduate medical curriculum and the residency program directors. Initially, some faculty members expressed concerns that a focus on recognizing conservative beliefs, spirituality and religious values could be used to mandate curricular changes --- for example, forcing a reconsideration of topics such as immunization, reproductive health or even evolution. Ultimately, the vast majority of faculty members understood that students were not calling for changes in lecture topics or how science is presented; rather, the message of the survey was that students holding differing political or religious views should always be treated with respect. Students and faculty members also heard about the importance of respectful treatment of individuals from racial or ethnic minority groups, the GLBT community, and women and respectful treatment of patients and other individuals who do not speak English or who are poor or uninsured. Faculty members who heard these results appeared to gain a greater appreciation of this broader definition of diversity, as seen through their students’ eyes.

The medical school has taken several actions since the survey results were released. First, the School’s Diversity Mission Statement and the teacher-learner contract, a document that outlines the expectations and shared responsibilities of students and teachers, were modified to include respect for “political values and beliefs”. In addition, the following statement was added to both documents: “In all educational, research and clinical care settings, the school will welcome and respect all religious, spiritual and political beliefs and will welcome and respect patients and others who are poor, disadvantaged, uninsured or non-English speaking”. Lastly, the medical school has developed an anonymous and confidential online professionalism reporting system that will permit students to report incidents of exemplary or poor professional behavior by residents or faculty. Taken together, these three changes signify a renewed commitment to improving the campus diversity climate and ensuring a safe and vibrant learning environment for all students.

## Conclusion

This study, though limited by a low response rate, supports previously published studies indicating that diversity climate issues exist at American medical schools. Our data also highlight that individuals with conservative religious and political views are often targets of disparaging remarks and offensive behaviors. This is a novel finding. Lastly, our study emphasizes the importance of assessing a medical school’s climate and working definition of diversity from a student body perspective.

## Competing interests

The authors declare that they have no competing interests.

## Authors’ contributions

JD designed the survey instrument, collected survey data, aided in data analysis and drafted the manuscript. LC aided in survey design, data analysis and interpretation. MV performed the statistical analysis and aided in drafting the manuscript. SL conceived of the study, aided in survey design and data collection, conducted the data analysis and aided in drafting the manuscript. All authors read, edited and approved the final manuscript.
